# Conceptual models for implementing solution-oriented team science in large research consortia

**DOI:** 10.1017/cts.2021.802

**Published:** 2021-06-14

**Authors:** Leslie C. Thompson, Kara L. Hall, Amanda L. Vogel, Christina H. Park, Matthew W. Gillman

**Affiliations:** 1 Environmental influences on Child Health Outcomes, Office of the Director, National Institutes of Health, North Bethesda, MD, USA; 2 Division of Cancer Control and Population Sciences, National Cancer Institute, Rockville, MD, USA; 3 Office of Policy, Communications and Education, National Center for Advancing Translational Sciences, Bethesda, MD, USA

**Keywords:** Conceptual models, solution-oriented research, transdisciplinary science, team science, large research consortia

## Abstract

Large translational research initiatives can strengthen efficiencies and support science with enhanced impact when practical conceptual models guide their design, implementation, and evaluation. The National Institutes of Health (NIH) Environmental influences on Child Health Outcomes (ECHO) program brings together data from 72 ongoing maternal–child cohort studies – involving more than 50,000 children and over 1200 investigators – to conduct transdisciplinary solution-oriented research that addresses how early environmental exposures influence child health. ECHO uses a multi-team system approach to consortium-wide data collection and analysis to generate original research that informs programs, policies, and practices to enhance children’s health. Here, we share two conceptual models informed by ECHO’s experiences and the Science of Team Science. The first conceptual model illuminates a system of teams and associated tasks that support collaboration toward shared scientific goals. The second conceptual model provides a framework for designing evaluations for continuous quality improvement of manuscript writing teams. Together, the two conceptual models offer guidance for the design, implementation, and evaluation of translational and transdisciplinary multi-team research initiatives.

## Introduction

Scientific organizations are increasingly employing large research consortia in efforts to produce innovative solutions to challenging health problems [1]. Many of these consortia involve multi-team systems, i.e., multiple teams working cooperatively to achieve shared superordinate goals [2]. The relationships among the numerous tasks needed to achieve scientific goals become more challenging to coordinate as consortia grow in size and work complexity [3]. Thus, designing teams and activities to better organize collaborative workflows is vital to enhancing consortium productivity [4,5].

Several existing conceptual models offer insight into the complexities of implementing or evaluating team-based research. Hall *et al.*’s Four-Phase Model of Transdisciplinary Team-based Research offers guidance to support team processes at each stage of a research initiative, from developing ideas to implementing complex team-based research toward translational applications [6]. Turner *et al.*’s Multi-Team System Effectiveness Model illuminates the process-level topology of teamwork, taskwork, performance, and value to inform consortia functioning [7]. Trochim *et al.*’s evaluation model for the Transdisciplinary Tobacco Use Research Center Initiative emphasizes quality improvements that are aimed at enhancing collaborative processes [8]. Luke *et al.*’s Translational Science Benefits Model provides evaluators with a framework for assessing the benefits of clinical research beyond bibliometric outcomes of publications, including policy, economic, or public health advancements [9]. While these models offer insight into the performance of research consortia, fewer conceptual models exist that also inform the design of consortium operations. Considering the substantial financial investments in large research consortia [10], conceptual models tailored to guiding their design, implementation, and evaluation can strengthen efficiencies to support science with enhanced impact, thus offering a better return on investment.

The Environmental influences on Child Health Outcomes (ECHO) program is one example of a large-scale, high-investment multi-site research consortium. The mission of ECHO is to enhance the health of children for generations to come. Launched by the National Institutes of Health (NIH) in 2016, the primary scientific goal of ECHO is to answer solution-oriented research questions about how a broad array of early environmental exposures influence common health outcomes throughout childhood and adolescence.

Since its inception, ECHO investigators have developed multi-site collaboration strategies to help promote efficiencies that often challenge large consortia [11]. In this article, we share two conceptual models that reflect ECHO’s experiences and key concepts from the Science of Team Science. These models aim to provide funders, practitioners, and evaluators of large research consortia with practical approaches to inform the design, implementation, and evaluation of large translational and transdisciplinary research consortia. Toward helping readers interpret and use these models, Table 1 provides a list of definitions.

**Table 1.** Definitions

Key term	Definition
Common Data	Similar or identical data elements collected at multiple study sites that are made available for use in consortium-wide analyses
Cores and Centers	Teams of investigators that coordinate administrative or operational activities, provide data management and analysis support, or supply various types of technical expertise to a research consortium
Continuous Quality Improvement	Evaluative monitoring of progress toward goals to iteratively enhance system operations, work environments, processes, outputs, and outcomes [29]
End user Stakeholders	Organizations that use research results to develop programs, policies, and practices
End user Stakeholder Needs	Research evidence needs that, when addressed, allow end user stakeholders to enact solutions
Investigator	A researcher on a scientific project
Multi-Team System	Multiple teams working cooperatively with each team contributing to achieve shared superordinate goals [2]
Quality	The degree of excellence inherent to system operations, work environments, processes, outputs, or outcomes [35]
Research Consortium	A collective of independent research teams each contributing to shared scientific goals
Steering Committee	A committee of principal investigators and funding agency staff that govern the scientific direction of a consortium and make key operational decisions
Solution-oriented Research	A paradigm for framing scientific questions to produce study results that can directly inform programs, policies, and practices toward enhancing health [17]
Team Functioning	The emergence of (a) affective qualities, e.g., trust and psychological safety [3]; (b) group cognition, e.g., shared understanding of the collaborative research [3]; and (c) behavioral features, e.g., cohesiveness and effectiveness [24]
Team Science	Two or more investigators conducting scientific work together in an interdependent fashion
Transdisciplinarity	The integration of perspectives to produce scholarship that extends beyond the contributing investigators’ disciplines to yield innovative research [14]
Translational Research	The scientific process for translating laboratory or clinical observations into interventions that improve health [22]
Value	The degree of worth an individual, team, organization, or institution assigns to system operations, work environments, processes, outputs, or outcomes [35]
Writing Team	A team that forms around a research idea, develops the idea into an analysis proposal, incorporates stakeholder perspectives/needs, conducts analyses, and publishes the analysis results

## Rationale: The ECHO-Wide Cohort and Transdisciplinary Solution-Oriented Research

Efforts to solve complex societal challenges can benefit from large-scale research initiatives that work to shift paradigms, develop innovative technologies, and generate large datasets [12]. One such complex scientific challenge is to understand how the array of environmental exposures occurring during early human development influence health trajectories from childhood through adolescence. NIH launched the ECHO-wide Cohort – the keystone of ECHO’s observational research studies – to advance knowledge in this area of inquiry. The ECHO-wide Cohort is an omnibus comprising 72 ongoing maternal–child cohort studies, all of which predate ECHO. These cohorts span 158 study sites across 33 US states, Washington, D.C., and Puerto Rico. ECHO also funds cores and centers that provide the consortium with research activity coordination and data support.

The aim of the ECHO-wide Cohort is to bring together data and biospecimens from all these ongoing studies, under a common data collection protocol. As of 2021, the ECHO-wide Cohort data platform contains data on over 50,000 children and their families, which ECHO will make available to the research community as a national resource for studying child health. With a substantial sample size and participant diversity suitable for studying broad public health issues, investigators can use ECHO-wide Cohort data to address research questions that no single cohort, or even a few, could address alone.

In addition to bringing together data, ECHO brings together over 1200 investigators from a wide variety of disciplinary backgrounds, including maternal and child health, public health, clinical, epidemiologic, psychosocial, biochemical, computational, and other sciences to conduct transdisciplinary research. Sometimes referred to as convergence research [13], transdisciplinarity refers to the integration of perspectives to produce scholarship that extends beyond the contributing investigators’ disciplines to yield innovative research [14]. Such transdisciplinary approaches have the potential to produce holistic findings with relevance to public health interventions [14].

Transdisciplinary research also emphasizes the importance of engaging stakeholders throughout the scientific process to produce findings that better address stakeholder needs [15]. The ECHO program focuses on the up-front engagement of end user stakeholders, which include researchers who conduct intervention studies; patient- and community advocacy organizations; medical and public health professional societies; and local, state, and federal government agencies. Up-front end user engagement (a) allows investigators to frame their questions to address specific evidence needs and (b) increases the likelihood that end user stakeholders will use the results to drive public health actions [16]. While conversations among investigators and end users can happen directly, consortia can also glean these evidence needs from end user stakeholders’ publications that call for specific research actions.

Solution-oriented research is a paradigm that urges investigators to frame their research questions so study results can directly inform actions that enhance health [17]. While the paradigm traditionally applies to intervention studies, it also applies to observational studies, which can directly inform intervention trials, health policies, and clinical practice guidelines. Examples of solution-oriented research are trajectory analyses to help pinpoint the timing of critical or sensitive periods during development [18]. Research on biological mechanisms can inform prevention strategies [19]. Studies that estimate the association of risk factor combinations with health outcomes may inform multicomponent interventions [20]. As individuals are often exposed to combinations of chemicals, mixture analyses that identify the sources of the most toxic chemicals can inform policies that mitigate exposure [21].

Readers may note overlap in the principles of solution-oriented research and translational research, which is the scientific process for translating laboratory or clinical observations into interventions that improve health [22]. Solution-oriented research, as we discuss it, is about framing research questions to directly address end user stakeholder needs within the broader translational research process.

Part of the vision for the ECHO-wide Cohort is to marry transdisciplinarity and solution-oriented research. ECHO conceptualizes solution-oriented research at the intersection of three constituent elements: (a) end user stakeholder needs; (b) research ideas driven by investigator passion; and (c) available common data, which in the case of ECHO includes high-quality data collected at multiple study sites and integrated on a shared data platform to foster collaboration. Supplementary Fig. S1 provides a Venn diagram of this concept. ECHO uses a multi-team system designed specifically to address each of these three elements during its production of research.

In the next sections, we share two conceptual models for solution-oriented team science for use in large research consortia. The first conceptual model illuminates a system of teams and associated tasks that support collaboration toward shared scientific goals. The second conceptual model offers a framework of team functioning, with variables to consider for continuous quality improvement of the system’s manuscript writing teams. Together, the two conceptual models offer guidance for the design, implementation, and evaluation of transdisciplinary team-based research initiatives. See the Supplementary Material for videos that build each of these models one feature at a time.

## Conceptual Model 1: Multi-Team System Blueprint for Generating Solution-Oriented Research

**Fig. 1.**
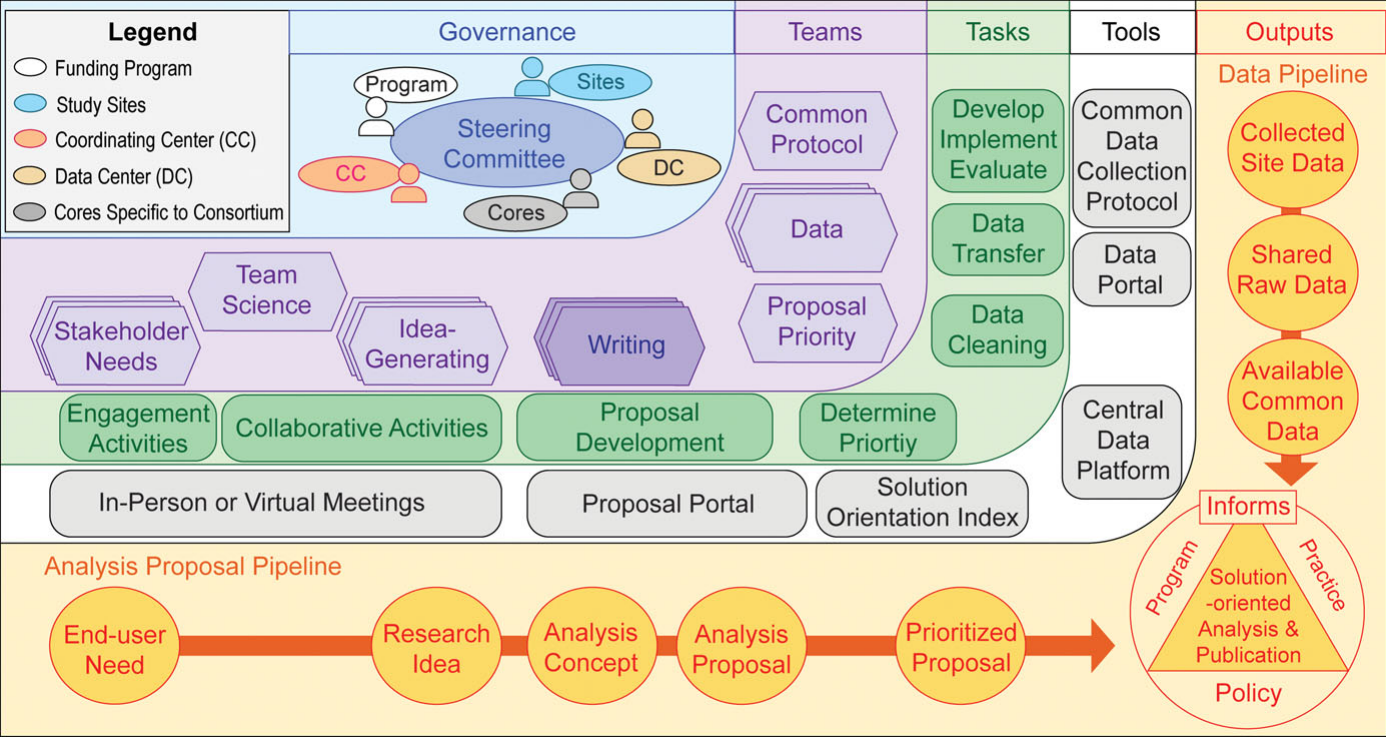
Multi-team system blueprint for generating solution-oriented research (reviewed in the Supplementary Video for Model 1).

Effective multi-team systems use carefully designed workflows to align each team’s outputs with shared goals [2]. To address such a need, the first conceptual model (Fig. 1) offers a blueprint for generating solution-oriented research in a multi-team system. The model is organized into five dimensions, each depicting a generalized element of ECHO’s multi-team system design: (1) governance; (2) teams; (3) tasks; (4) tools; and (5) outputs. These dimensions drive two intersecting pipelines: one for developing analysis proposals from end user needs and research ideas (bottom horizontal in Fig. 1) and one for creating a common dataset available for consortium-wide analyses (right vertical in Fig. 1). Here, we describe each of the five dimensions of the model.

### Governance, Teams, Tasks, and Tools

#### Governance

In the upper left corner of Fig. 1, the first dimension of the model highlights the primary governance structure, a steering committee comprised of principal investigators from each study site, center, and core from the consortium, and at least one official from the funding program. Together, they share responsibility for governing the scientific direction of the consortium and making key operational decisions. For ECHO, the funding program in the model reflects the ECHO Program Office at NIH. The study sites represent ECHO’s cohort awards. The coordinating center and data center correspond to ECHO’s Coordinating Center and Data Analysis Center. The cores specific to the consortium in the model reflect ECHO’s Person-Reported Outcomes Core, Human Health Exposure Analysis Resource, and Genetics Core.

#### Teams

Members of ECHO – steering committee members and other consortium investigators – organize into a system of teams shown as hexagons in the second dimension of the model. These teams deal with the details of the consortium’s policies, operations, and science and report to the steering committee for decision-making. In the model, each team derives its name from its task or focus in the system. In some cases, there can be multiple teams, indicated by stacks of hexagons. For example, multiple stakeholder needs teams may each engage with distinct types of end users like patient advocacy organizations or medical professional societies.

#### Tasks and tools

Teams use a range of tasks and tools to implement their scientific work. The model shows these tasks and tools as rounded quadrilaterals in the third and fourth dimensions of the model, respectively. Some examples of tasks include engagement with stakeholders, collaboration across teams, analysis proposal development, implementing a common data collection protocol, and data transfer. A variety of tools support these tasks. For example, in-person and virtual meetings support engagement and collaborative activities. A proposal portal provides an online environment for developing analysis proposals. Tools in the data infrastructure include electronic mediums for data collection (common data collection protocol), remote data entry and transfer (data portal), as well as cloud-based data integration and analysis (central data platform).

### Outputs: Analysis Proposal and Data Pipelines

#### Outputs

Consortium outputs, shown as circles in the outer dimension, move along the analysis proposal pipeline (bottom) and a data pipeline (right). While the outputs build on each other progressively along each pipeline, they also function interdependently. For example, data analyses require the availability of common data and research ideas may inform new data collection. The pipelines ultimately serve to enable the production of solution-oriented publications that are intended to inform programs, policies, and practices (bottom right).

#### Analysis proposal pipeline

The consortium’s analysis proposal pipeline flows along the bottom of the model (Fig. 1). Teams involved with identifying stakeholder needs can organize systematic end user engagement activities upfront. These activities might include reviewing white papers or annual reports to glean end user needs or inviting end users to present evidence needs to the consortium. Next, a “team science” team designs collaborative activities to help idea-generating teams develop research ideas the consortium can address with its available common data. These activities can include mini-hackathons with idea-generating team members from different disciplines organized into ad-hoc small groups. To prepare, the team science team collaborates with stakeholder needs teams and the consortium’s data center to offer information to small group participants about end user needs and available common data.

Next, writing teams form around research ideas, develop them into analysis concepts, and submit them through an online proposal portal run by the coordinating center. In ECHO, investigators use discussion boards within the portal to supply critical feedback to strengthen analysis concepts. Then writing teams develop their analysis concepts into more detailed analysis proposals, considering end user needs to frame solution-oriented research questions. As part of the analysis approval process, a proposal priority team uses a solution orientation index (Supplementary Table S1) to identify high-priority analysis proposals. In ECHO, a Publications Committee fulfills this prioritization and conducts manuscript review before writing teams submit their articles to scientific journals. If the committee designates an analysis proposal as a high priority, ECHO’s Data Analysis Center allocates additional resources to help speed the cleaning of data needed for the analysis, while the Coordinating Center provides additional support for organizing virtual writing team activities.

#### Data pipeline

The consortium’s data pipeline moves outputs along the right side of the model in the outer dimension, beginning with the collection of common data at study sites and ending in the development of a large-scale common dataset. To start, a common protocol team oversees development, implementation, and evaluation of the consortium’s common data collection protocol that study sites use to guide common data collection. ECHO uses a large omnibus protocol – the ECHO-wide Cohort Data Collection Protocol [23] – specifying essential and recommended data elements to collect according to study participant life stage. Next in the model, data teams assist study sites with the transfer of raw data, as well as data cleaning. In ECHO, this includes harmonizing large amounts of data collected during each individual cohort study for years before the program’s launch, as well as evaluating data quality and completeness. Within the tools dimension of the model, the data center houses the cleaned common data on their central platform, making them available to investigators for consortium-wide analyses approved by the steering committee.

When implemented, a consortium can evaluate the approach outlined in Model 1 to help identify challenges and intervene appropriately to improve quality. To this end, ECHO’s Steering Committee sets annual operational objectives for its analysis proposal and data pipelines. The Coordinating Center and Data Analysis Center collect indicator data for these objectives and populate a monitoring dashboard. ECHO recently set up a Program Evaluation and Mentoring Working Group to periodically review the dashboard, evaluate successes and challenges, and provide mentoring opportunities among investigators to share successful implementation strategies. The working group reports findings to the Steering Committee so ECHO can consider consortium-level interventions.

Team-level evaluation strategies can also enhance the success of the multi-team system approach from Model 1. For example, the model culminates with writing teams – the darker shade hexagon stack – generating solution-oriented analyses and publications (bottom right), developed at the intersection of end user needs, research ideas, and the consortium’s available common data. Each writing team works to integrate multiple disciplinary, experiential, and practical perspectives from their members, resulting in transdisciplinary products that, when successfully applied, can advance the science in new directions. In the next section, we offer a conceptual model for continuous quality improvement of manuscript writing teams from Model 1.

## Conceptual Model 2: Writing Team Functioning in Solution-Oriented Research

**Fig. 2.**
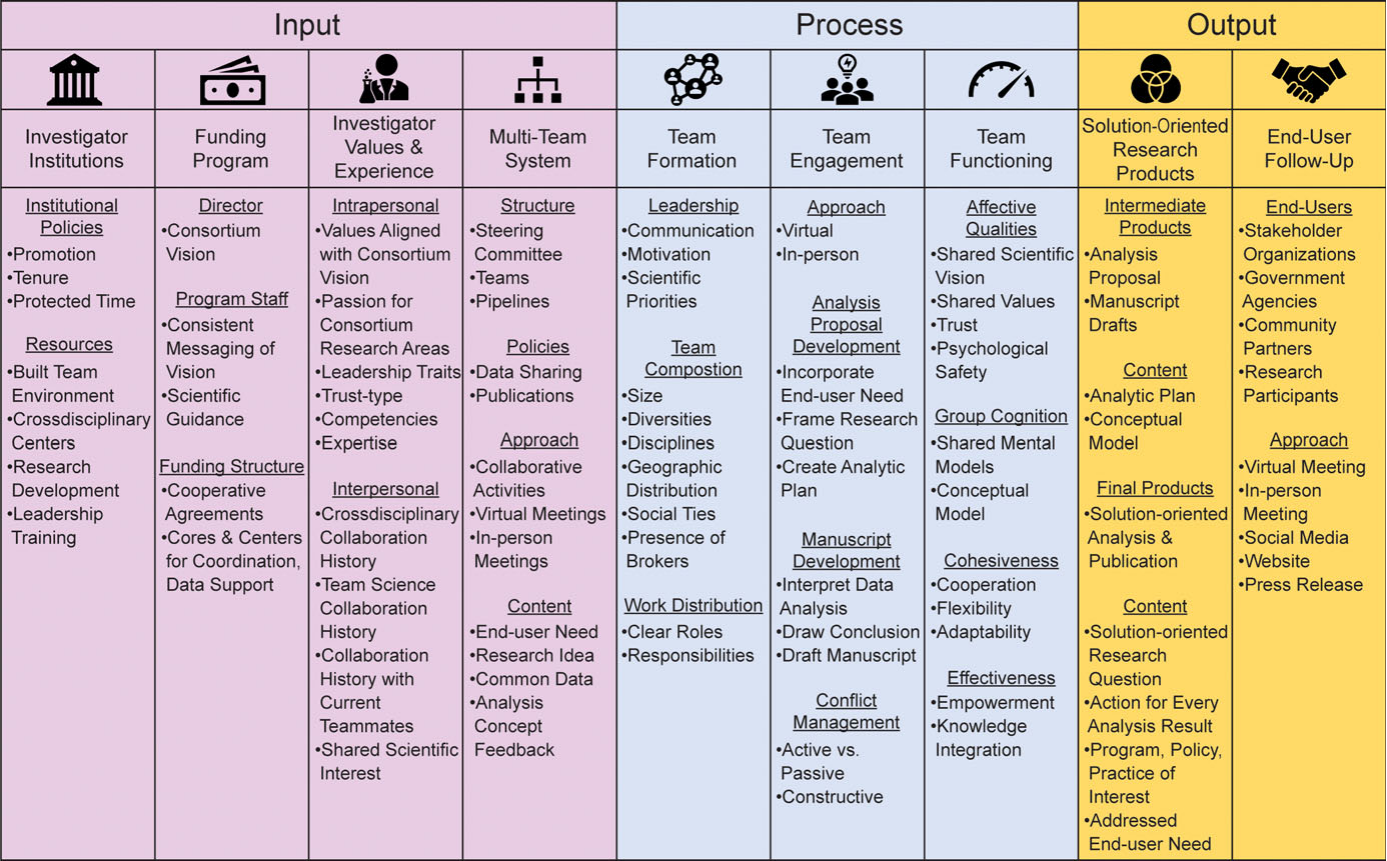
Writing team functioning in solution-oriented research (reviewed in the Supplementary Video for Model 2).

To capitalize on the potential benefits of the multi-team system in Model 1, writing teams must maximize effective functioning, which is the emergence of (a) affective qualities, e.g., trust and psychological safety [3]; (b) group cognition, e.g., shared understanding of the collaborative research [3]; and (c) behavioral features, e.g., cohesiveness and effectiveness [24]. The second conceptual model (Fig. 2) focuses on explicating writing team functioning in solution-oriented research within the context of the multi-team system depicted in Model 1. Input–process–output frameworks [25], like the one presented in Model 2, are ideal for continuous quality improvement – described in more detail below.

### Inputs, Processes, and Outputs

#### Inputs

Writing team inputs involve investigator institutions, the funding program, investigator values and experiences, and the multi-team system from our first model. Investigator institutions can influence collaboration by creating policies and providing resources that support faculty involvement in team science initiatives [3]. The funding program includes a director who promotes a vision for the consortium, program staff that manages the program to achieve the vision, and funding structures such as cooperative agreements in which the funder assists the awardees in reaching consortium goals. Investigator values and experience can influence team processes [26]. The values and experiences can each be divided into two categories. The first is intrapersonal, e.g., their passion for the consortium’s overall scientific vision. The second is interpersonal, e.g., their history of collaboration. The final input listed is the multi-team system from Model 1 because writing teams integrate the system’s outputs.

#### Processes

Writing teams engage in three key teamwork processes: team formation, team engagement, and team functioning. Team formation can involve (a) leadership [3], i.e., how the leaders address team member motivation or help to guide the process of developing the team’s scientific priorities [6]; (b) team composition, i.e., team size, diversity, disciplines, and presence of brokers [3]; and (c) work distribution, i.e., how the team establishes roles and responsibilities [27]. Team engagement refers to the virtual or in-person approaches that writing teams implement to generate solution-oriented research, including development of analysis proposals and manuscripts. To develop their solution-oriented analysis proposal, a writing team must frame a research question that addresses an end user need and create an analytic plan. Team engagement involves close attention to conflict management [24]. Over time, high-functioning teams cultivate affective qualities, group cognition, cohesiveness, and effectiveness [6].

#### Outputs

Solution-oriented research products – analysis proposals and publications – and end user follow-up are the two main outputs of writing teams. These publications typically address end user needs thereby informing programs, policies, and practices. After the publication of the manuscript, a writing team can follow-up with end users in person, virtually, or on social media to disseminate findings, as well as promote solutions [28].

Model 2 emphasizes the role that writing teams play in our multi-team system from Fig. 1. Consortia can engage in continuous quality improvement to overcome challenges writing teams face while generating their final research products. Continuous quality improvement is evaluative monitoring of progress toward goals to iteratively enhance system operations, work environments, processes, outputs, and outcomes [29]. One approach is to examine the team processes involved in creating intermediate products – analysis proposals or manuscript drafts [6,8]. For example, revisions to a conceptual model over drafts of both intermediate and final products reflect how a writing team’s group cognition progressed over time. Authorship of edits in these drafts can help document who contributes different disciplinary or stakeholder perspectives so that evaluators can better understand how their perspectives influenced the evolution of the writing team’s shared scientific vision. Such evidence could inform consortium-wide strategies to enhance writing team processes. The Publications Committee in ECHO offers feedback on quality improvement to writing teams when reviewing analysis proposals and helps monitor timelines to ensure writing teams stay on track.

Writing teams bring together the outputs from Model 1. As such, the broader multi-team system in Fig. 1 offers a consortium many variables that can influence writing team outputs to consider for continuous quality improvement. As an example, in the Supplemental Material, we pose a hypothetical evaluation question about the extent to which end user engagement during analysis proposal development adds value to a writing team’s solution-oriented research products. Supplementary Fig. S2 shows pathways of influence among the inputs, processes, and outputs in Fig. 2. Assessing the value proposition of up-front end user engagement would produce findings with ramifications for how a consortium should engage end users during future analysis proposal development. In ECHO, the Stakeholder Engagement Working Group and Team Science Working Group are well positioned to conduct this type of evaluation during end user follow-up to enhance up-front engagement strategies.

## Discussion

Drawing on lessons learned from ECHO and the Science of Team Science, we developed two conceptual models to guide the design, implementation, and evaluation of large consortia working to produce high-impact public health research. This paper addresses the need for practical conceptual models to help large research consortia effectively navigate transdisciplinary, solution-oriented, multi-team research collaborations. The models fill this need by offering a blueprint for a multi-team system approach and highlighting the key variables of writing team functioning. Conceptual models such as these, which codify organizational schemes, define processes, and present foci for quality improvement efforts, can help strengthen return-on-investment for multiyear, high-budget research initiatives [8].

The literature that documents the value of large transdisciplinary team science collaborations often mentions the integral nature of stakeholder engagement to achieving actionable research findings [30]. Our models build on this literature by introducing solution-oriented research as an organizing principle for designing these large initiatives [31]. Other novel features include examples of tools that teams use to support tasks, as well as combining the taskwork of solution-oriented research with the teamwork of science teams. Overall, these are the first models to combine design features with the potential for guiding evaluation.

The first model shows a blueprint for designing a multi-team system based on the goals of solution-oriented research. As suggested, leaders can inform the design of an effective multi-team system by first conceptualizing a consortium’s overarching scientific goals, range of expertise needed to adequately address the science, as well as potential breadth of data and end user involvement. Then, consortium members can specify the requisite teams, tasks, tools, and output workflows needed to operationalize the goals.

The second model highlights the practical activities to incorporate solution-oriented research questions into the taskwork of science teams. These activities include considering end user perspectives to identify actionable research needs [16]. Model 2 also underscores key teamwork processes of science teams related to team formation, engagement, and functioning [3], while placing the processes in the context of writing teams working in a multi-team system conducting solution-oriented research.

A key concept likely to remain consistent as other consortia apply our models is the engagement of end user stakeholders throughout the scientific process. However, the form and extent of engaging end users will vary among consortia. In addition to community advocacy organizations, health professionals, and policy makers, other scientists can be end user stakeholders. For example, clinical trialists can be end users of observational research. The same principles apply among researchers carrying out basic, preclinical, or clinical studies so that one translational stage informs the others more directly [32].

While findings from a structured evaluation are forthcoming, ECHO has already shown early success. While developing the ECHO-wide Cohort data platform, ECHO has produced over 600 publications since 2016 [33]. These papers mostly comprise analyses of existing data from individual awards, as well as 27 consortium-wide collaborations on new methodologies and off-platform analyses involving data use agreements. ECHO embodied the solution-oriented team science approach to respond quickly to the COVID-19 pandemic [34]. The response included awarding competitive supplemental funding to teams of ECHO investigators for time-sensitive COVID-19-related research. To help standardize national research on the consequences of the pandemic, ECHO rapidly developed COVID-19 questionnaires and shared them with the broader scientific community. The consortium also incorporated the COVID-19 questionnaires into the ECHO-wide Cohort Data Collection Protocol and used the protocol’s online, mail, and phone surveys, as well as at-home biospecimen collection kits, to continue data collection during the pandemic. As a result, several COVID-19-focused data analyses and manuscripts are underway.

The ultimate value of the two models we present will rely on other consortia adopting, adapting, assessing, and refining them. Consortia other than ECHO could use the models to guide the implementation of their research goals, adjust the models as needed, and report on the results of any changes. Depending on the goals of the program, the teams and tasks may be different from those in these models. If the models inform continuous quality improvement, consortia can share information about their effectiveness to improve their multi-team system approach.

In conclusion, the solution-oriented team science models presented here offer frameworks to guide translational and transdisciplinary multi-team consortia through design, implementation, and evaluation. In combination with sound scientific goals, large consortia need sophisticated workflows to see maximum return on investments, including research productivity and potential for public health impact. Without sufficient attention to these operational features, large research initiatives may produce science that differs very little from that of a collection of individual research grants, but at a much higher cost. On the other hand, consortia that incorporate the features of these models have the potential to accelerate understanding of complex public health problems while pointing to effective solutions.

## Supplementary material

For supplementary material accompanying this paper visit https://doi.org/10.1017/cts.2021.802. click here to view supplementary material
