# Supplementary material for: Conceptual models for implementing solution-oriented team science in large research consortia
Source: J Clin Transl Sci. 2021 Jun 14;5(1):e139. doi: 10.1017/cts.2021.802 (PMC8327547; doi:10.1017/cts.2021.802)

**Supplement for “Conceptual Models for Implementing Solution-Oriented Team Science in Large Research Consortia”**

Leslie C. Thompson^1^, Kara L. Hall^2^, Amanda L. Vogel^3^, Christina H. Park^1^, Matthew W. Gillman^1^

^1^Environmental influences on Child Health Outcomes, Office of the Director, National Institutes of Health, Rockville, MD 20852; ^2^National Cancer Institute, Rockville, MD 20850; ^3^National Center for Advancing Translational Sciences, Bethesda, MD 20817

Correspondence:

Leslie C. Thompson, Ph.D.

Environmental influences on Child Health Outcomes

National Institutes of Health, Office of the Director

11601 Landsdown Street | Office 03D12

North Bethesda, MD 20852

[p] 301-435-5239

leslie.thompson@nih.gov

**Supplement Introduction**

This supplement includes figures and a table that expands on the two conceptual models in the primary paper. These highlight the three conceptual elements of solution-oriented research; a solution orientation index that consortia can use to identify priority analyses, and key pathways in writing teams from our solution-oriented multi-team system.

There is also a video in the Supplemental Materials that builds Model 1—from the main paper—one feature at a time.

**Three Elements of Solution-Oriented Research**

ECHO conceptualizes solution-oriented research at the intersection of three constituent elements: a) end-user stakeholder needs, *i.e.*, evidence needs end users identify that, if filled, would drive enacting solutions b) research ideas driven by scientific areas of investigator passion, and c) available common data, *i.e.*, high quality data elements collected at multiple study sites in a consortium, in some cases transferred to a shared location or platform to foster collaboration. Supplemental Figure S1 is a Venn diagram of this conceptualization. The diagram highlights feasible research in the overlap between research ideas and the common data resource, as well as potential solution-oriented research in the overlap between end-user needs and the common data resource.

**Solution Orientation Index**

The Solution Orientation Index in Supplemental Table S1 is similar to ECHO’s Big Win Impact Scale, which allows the ECHO Publications Committee to assess an ECHO-wide Cohort analysis proposal for solution orientation, innovative capacity, and analytic rigor.

**Key Pathways in Writing Teams**

Those interested in evaluating a consortium can measure how well inputs, processes, and outputs from the second conceptual model in the main paper relate to one another. As the multi-team system outlined in the first conceptual model influences writing teams in various interacting ways, all aspects of the system present potential subjects for evaluation aimed at continuous quality improvement. In Supplemental Figure S2, we pose a hypothetical evaluation question about the extent to which end-user engagement during analysis proposal development adds value to a writing team’s solution-oriented research products.

We developed the linkages between the inputs, processes, and outputs by first considering the outputs and thinking about what their key influences might be. For example, a solution-oriented analysis and publication would need to follow an analysis proposal with a solution-oriented question. We used these two intermediate outputs to conceptualize two pathways through the model. Next, we are proposing that writing teams engage end users during analysis proposal development, which would influence the end-user need input. Writing teams also form around the inputs of a research idea. As a third related input, shared scientific interest could also be essential to writing team formation and all three inputs may influence the disciplines reflected in the writing team membership. The end-user need drives a writing team process pathway that links to the solution-oriented research question and the research idea propels a pathway that shapes the analytic plan, conceptual models, and eventually the analysis proposal.

As the purpose of the logic model in Supplemental Figure S2 is to support evaluation of end-user engagement, evaluators could start by querying end users to gauge the benefits and burdens of the consortium’s engagement strategies. From there, the influencing factors for each model element clarify a potential evaluation strategy. For example, different disciplines have different conceptualizations of compelling research ideas, data, and methods. To examine the influence of investigators’ home disciplines on their experiences in the writing teams, evaluators could survey writing team members on the extent to which the research idea, shared scientific interest with other members, and the identified end-user need inspired them to join the writing team and influenced the writing team process and outcomes. Similarly, survey questions could address varieties of pathways in the model. In another example, to look at the impact of end-user engagement, evaluators could use in-depth interviews of writing team members and end users to explore how their interactions helped incorporate end-user needs into the research analysis proposal, refinement of the research question, and interpretation of analysis results.

**Supplemental Figure S1: Three Elements of Solution-Oriented Research**


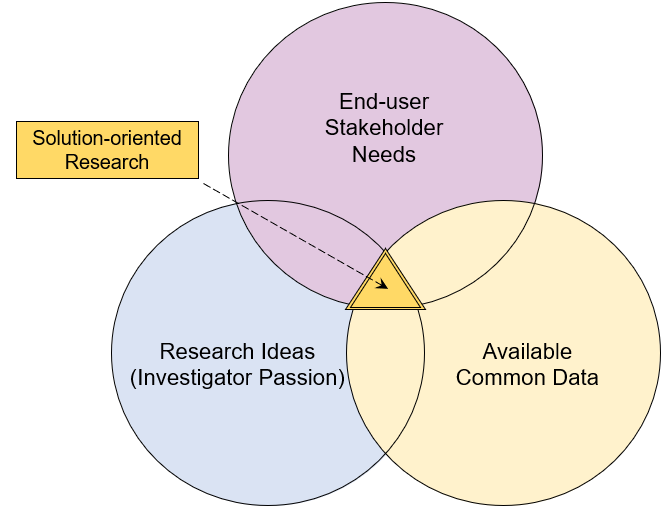


**Supplemental Table S1. Solution Orientation Index**

| **Criterion** | **Score (1-5)** |
| --- | --- |
| Aligns with Specific End-user Need^1^ |  |
| Solution-oriented Research Question^2^ |  |
| Innovation^3^ |  |
| Capitalizes on Unique Features of Consortium^4^ |  |
| Rigorous Analytic Plan^5^ |  |
| Overall Score^6^ |  |
| 1. The proposal identifies and addresses a specific need conveyed by an end-user stakeholder group 2. The proposal frames a research question such that the main study result will inform a specific intervention trial, clinical or public health practice, or policy (including resource allocation) 3. The analysis proposal uses novel concepts, approaches, or methodologies 4. Factors to consider include:    1. Sample size large enough for confidence in results, *i.e.*, narrow enough confidence interval to rule in or out important biological, clinical, and/or public health effects    2. Sample diversity in race/ethnicity, socioeconomic status, and/or geography 5. Conceptual model and analysis allow as much causal inference as possible 6. Solution orientation score (1-5): 1=exceptional, 2=excellent, 3=good, 4=fair, 5=low | |

**Supplemental Figure S2:**  **Key Pathways in Writing Teams**


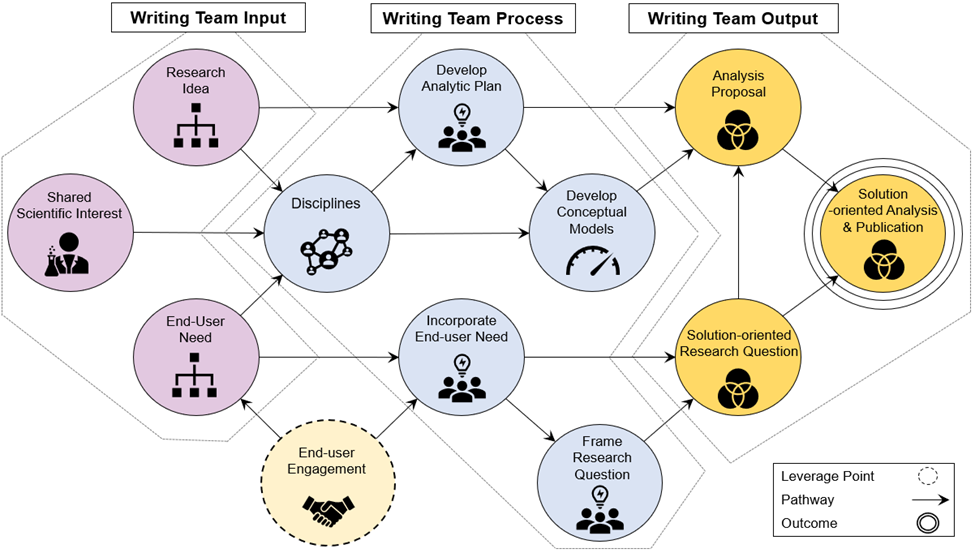

Supplement: Supplementary file 1 [file S2059866121008025sup.zip › S2059866121008025sup003.docx]
